# Supplementary material for: Chromothripsis during telomere crisis is independent of NHEJ, and consistent with a replicative origin
Source: Genome Res. 2019 May;29(5):737–49. doi: 10.1101/gr.240705.118 (PMC6499312; doi:10.1101/gr.240705.118)
Supplement: Supplemental Material [file supp_gr.240705.118_Supplemental_file_1.zip › contigs/annotated_contigs/DB38/contig.4.DB38_length_632_mean_cov_7.17246835443.docx]

**DB38_length_632_mean_cov_7.17246835443**

```
TAGAGAAGAGGCATACCTTAGAAGAAACACTATTGAAAGGCCATGTTTGTTTGTTTATGTAGGTCTTCTAAAACAAGCTGCCATCTTTA
 >chr11:132274600-132274910 + E=8e-175 p=4e-03
ATACTCATTTCTCAATCTCAGTCCAAAAACACTTTCTCTAAGTAAAATGAATGAGGAAATTCAGTTTCCATTTTACTTTTAGTTTAGTT

TAGCCAAGGAGTTTGACAAAAATCGCTTAAAAAAATCTTTTAAGGAATGCATTAAGGCTAAAAAAGCCCAAACCCACCATGAGAAAGCA

CATCACAGAAGGCCCTATATGTCCTTATTCTTTATTTTTTTA|T|TATTTATTATTTATTATTATTATTTAT|AA|ATAATATATATTA
                                           >chr3:164729551-164729579 + E=4e-05 p=0e+0
TTTATTATATATATTATAAATATATAT|AAATT|TATACTATGCAGCCATAAAAGAGGATGAGTTCATGTCTTTTGCAGGGATGTAAAT
0   >chr3:59200998-59201036 - E=3e-03  >chr11:132240561-132240809 + E=2e-137
GAATCTGGAAACCATCATTCTCAGCAAACTAACACAGGAACAGAAAACCAAACACCACATGTTCTCACTTATAAGTGGGAGCTGAACAA

CGAGAACACATGGACACCGGCAGGGAAACATTACACACCAGGGCCTGTCATGGGGTGGAGGGCTAGGGGAGGGACAGCATTAGGAGAAA

TGTCTAATGTAGATG
```
